# Automatic Multi-Stage Cold Forging of an SUS304 Ball-Stud with a Hexagonal Hole at One End

**DOI:** 10.3390/ma13225300

**Published:** 2020-11-23

**Authors:** Jong Bok Byun, Mohd Kaswandee Razali, Chang Ju Lee, Il Dong Seo, Wan Jin Chung, Man Soo Joun

**Affiliations:** 1 Engineering Research Institute, School of Mechanical and Aerospace Engineering, Gyeongsang National University, Jinju 52828, Korea; 200472@gnu.ac.kr; 2 Graduate School of Mechanical and Aerospace Engineering, Gyeongsang National University, Jinju 52828, Korea; mohdkaswandee@gnu.ac.kr; 3 Daedong Co. Ltd. 47-59, Dongbuk-ro 1109 beon-gil, Sangdong-myeon, Gimhae-si 50803, Gyeongsangnam-do, Korea; leecjnet@hanmail.net (C.J.L.); idm0070@naver.com (I.D.S.); 4 Department of Mechanical system Design Engineering, Seoul National University of Science and Technology, Seoul 01811, Korea; wjchung@snut.ac.kr

**Keywords:** high-strength material, stainless steel, flow stress characterization, non-isothermal analysis, cold forging

## Abstract

SUS304 stainless steel is characterized by combined tensile and compression testing, with an emphasis on flow stress at higher strain and temperature. The plastic deformation behavior of SUS304 from room temperature to 400 °C is examined and a general approach is used to express flow stress as a closed-form function of strain, strain rate, and temperature; this is optimal when the strain is high, especially during automatic multi-stage cold forging. The fitted flow stress is subjected to elastothermoviscoplastic finite element analysis (FEA) of an automatic multi-stage cold forging process for an SUS304 ball-stud. The importance of the thermal effect during cold forging, in terms of high material strength and good strain-hardening, is revealed by comparing the forming load, die wear and die stress predictions of non-isothermal and isothermal FEAs. The experiments have shown that the predictions of isothermal FEA are not feasible because of the high predicted effective stress on the weakest part of the die.

## 1. Introduction

In the coming era of electric and hydrogen cars, autopart industries must innovate in various ways. The forging industry, which has traditionally provided the materials used to make power transmission components, is facing major challenges. Plate forging and bulk sheet forming of metal parts, which can replace conventional heavy parts, of electric and/or electronic devices have been ongoing for two decades [1,2,3,4]. Additionally, the forging of high-strength and/or lightweight materials has been intensively researched [5,6,7,8]. New car-friendly materials and applications have emerged.

Stainless steel is difficult to cold forge because of its high strength and high-level strain-hardening. However, heat treatment is usually not required after cold forging. This enhances safety because the various defects associated with non-standard heat treatment are absent. Cold forging of stainless steels [9,10,11,12,13] is very attractive because it is difficult to lubricate optimally designed dies. These have been long-standing difficulties for application engineers and researchers of materials. Applications remain very limited; advances in die materials and lubricants lag behind those in high-strength cold forged SUS and high-strength energy saver wire (ESW) materials [5,7,14]. Stainless steels do not require heat treatment after cold forging, which enhances safety. Power transmission parts with defects caused by heat treatment can create serious problems, although no coating is required, which minimizes environmental contamination.

The isothermal assumptions for the relatively low-strength carbon steel SCHW10A [15], developed for automatic multi-stage cold forging, yield reasonably efficient finite element analysis (FEA) solutions to most cold forging processes, with a few exceptions, such as cold forging dominated by dynamic strain aging [16,17]. Premature die fracture is not a problem, and the material exhibits low flow stress and good forgeability. However, for successful cold forging of high-strength materials, die pressure and frictional stress must be minimized, and die design must consider material temperature softening, die shrinkage, and die fatigue life. For the ESW materials [7], the Bauschinger effect reduces die stress by 8%, and also lowers the forming load; these must not be neglected during the cold forging of high-strength materials because die fatigue life decreases exponentially as die stress increases. For example, a 6% reduction in die stress from 850 MPa at a stress ratio *R* = 0.1 of 20% WC-Co 20% [18] increases the fatigue life by about 72% from the initial 39,000 cycles. Cold forging of high-strength materials is characterized by relatively high global heating that is locally intense in the major deformation region, because over 90% of energy consumption is dissipated as thermal energy (via viscous heating). This increase in temperature drastically decreases flow stress, particularly at lower temperatures. Ishikawa et al. [19] performed an experimental and numerical study on the effects of temperature on elastic recovery during aluminum forging, using an axisymmetric backward extrusion process at room temperature, and emphasized the effect of the temperature rise on dimensional accuracy after ejection of forged aluminum alloys. However, the dependence of flow stress on temperature and strain rate was not considered. Byun et al. [20] found that the flow stress of SUS304 decreased by about 25% when heating from room temperature to 200 °C. Note that the temperature may exceed 400 °C during automatic, high-speed multi-stage cold forging [20]. The flow stress thermal characteristics of low strain-hardening materials (including aluminum and ESW materials), and of materials exhibiting dynamic strain can trigger instability under plastic deformation. Therefore, non-isothermal analyses emphasizing the effects of temperature on flow stress are essential for the optimal design of cold forging processes. Decreases in flow stress caused by viscous heating must be considered when evaluating the forgeability of high-strength materials. In general, an increase from room temperature to moderate temperatures decreases flow stress. Here, we evaluate the feasibility of automatic multi-stage cold forging of an SUS304 ball-stud both numerically and experimentally, with an emphasis on flow stress characterization.

## 2. Characteristics of the Flow Stress of SUS304

We studied SUS304 coils prepared for automatic multi-stage cold forging, which does not require post-forging heat treatment or surface coating. The chemical composition is shown in Table 1.

**Figure 1:**
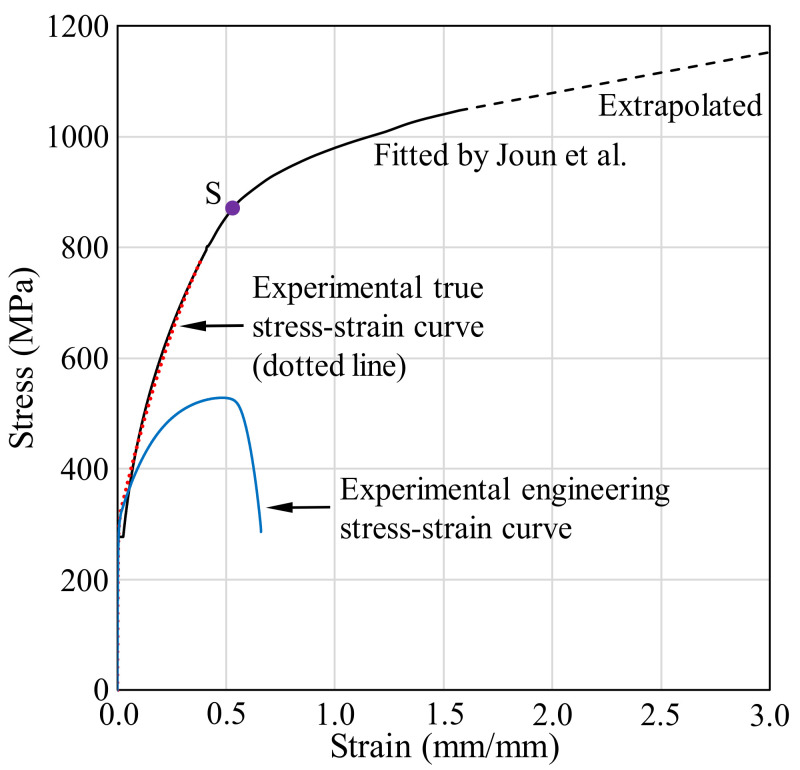
Tensile test curves and the flow stress curve of SUS304 at room temperature.

The material of a mechanically perfect, simple cylindrical bar, finite element model (gauge length = 50.0 mm, diameter = 14.0 mm) [21] was tensile tested at a low speed (0.17 mm/s). Compression tests were conducted at sample temperatures of 100, 200, 300, and 400 °C and strain rates of 1, 5, 10, and 20/s. Figure 1 shows the experimental data, i.e., the engineering stress–strain curve (the dotted line), the true stress–strain curve up to the necking point (the dashed line), and the corresponding flow stress curve (the solid line), as revealed by material identification technology [15]. The solid line was theoretically calculated, and the dotted line was extrapolated to meet the solid line.

**Figure 2:**
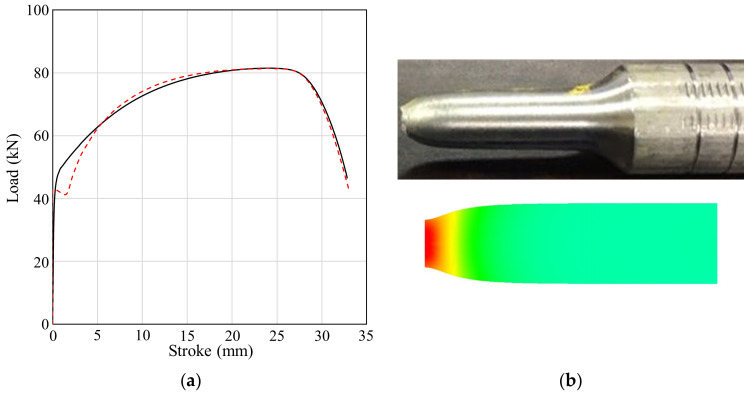
Comparison of the experimental and predicted tensile test results: (**a**) the tensile load–stroke; (**b**) the deformed shape.

**Figure 3:**
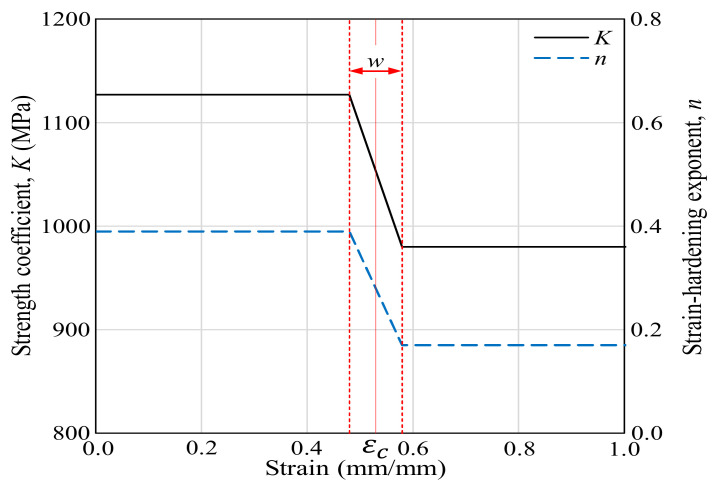
The strength coefficient and the strain-hardening exponent.

Figure 2 compares the experimental tensile load–stroke curve with the theoretical curve derived via FEA [15] using the flow stress of Figure 1. The results are in good agreement, so the theoretical flow stress of Figure 1 is valid. The engineering stress falls suddenly after the necking point; this is evident in the engineering stress–strain curve of Figure 1, which shows a drastic slope change in the flow stress–stroke curve around point S. A very similar flow stress curve for SUS304 was derived by Kweon et al. [22]. The flow stress curve in Figure 1 was fitted by the following double Hollomon law:

(1) $$\bar{\sigma }=K\left(\bar{\varepsilon }\right){\bar{\varepsilon }}^{n\left(\bar{\varepsilon }\right)}$$

where the strength coefficient and strain-hardening exponent, both of which are functions of strain, are formulated as the piecewise linear functions of Figure 3, where $\varepsilon_{c}=0.53$ is an intersection point of the double Hollomon law (denoted by S in Figure 1) and w=0.1 is the width of the strain that transits from the left to right Hollomon curve.

Note that the experimental tensile test of Figure 1 reveals typical strain-hardening behavior, and the double Hollomon law typically explains the flow stress of materials exhibiting such experimental tensile test behavior.

**Figure 4:**
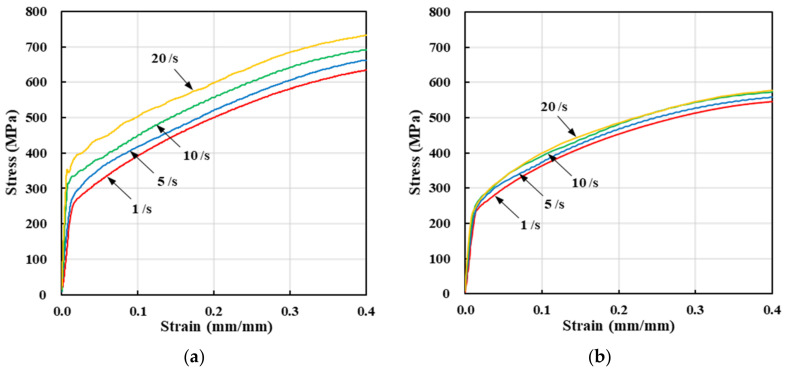

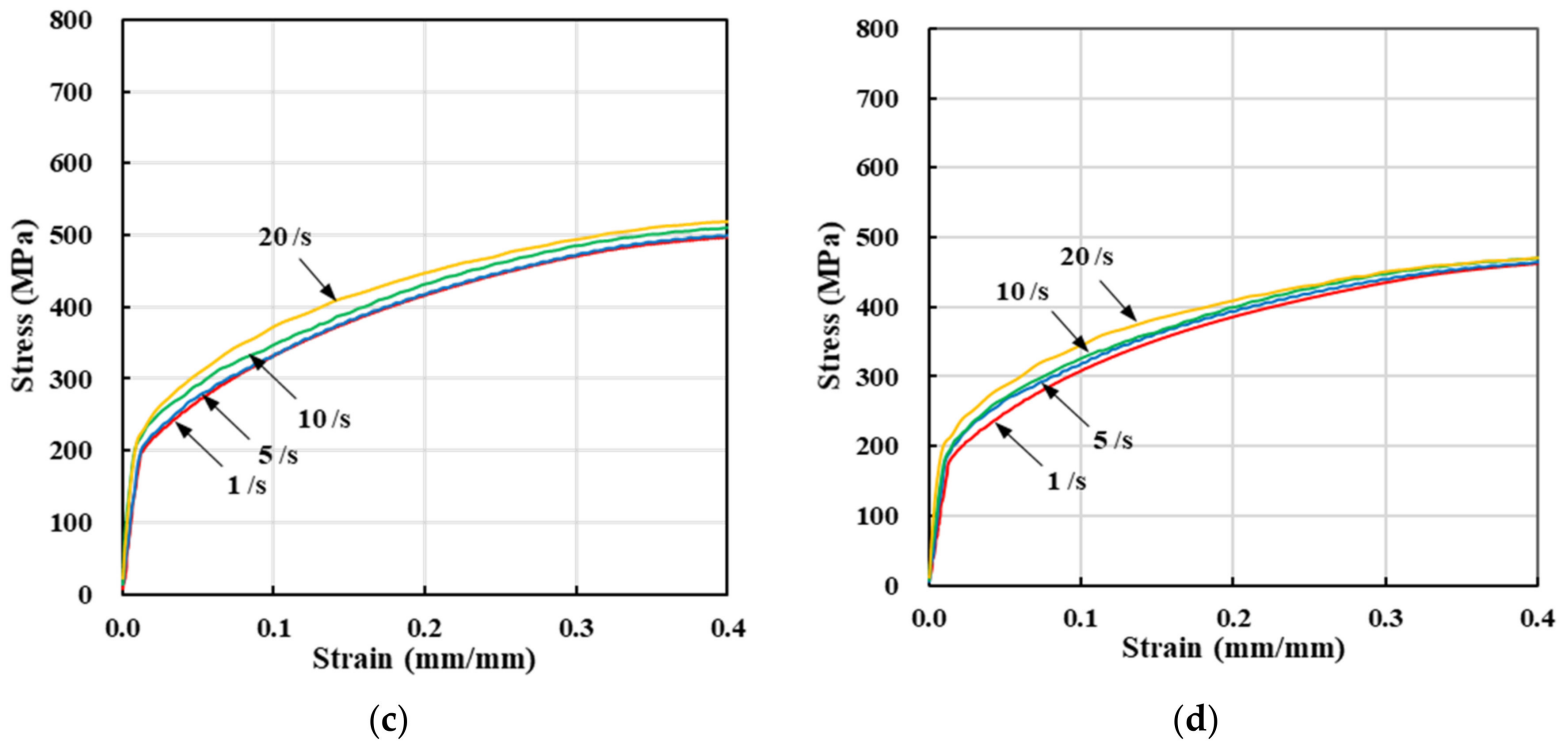
Flow stresses yielded by compression tests at various sample temperatures and strain rates: (**a**) 100 °C; (**b**) 200 °C; (**c**) 300 °C; (**d**) 400 °C.

Figure 4 summarizes the compression test data. The strain rate effect is remarkable at low temperature but decreases as temperature increases, then negligibly affects the flow stress. Notably, such flow behaviors are quite different from those of other materials. Figure 4 shows that the flow stress almost doubled when the strain increased from that of the yield point to 0.4 at 100 °C; similar phenomena were observed at 400 °C, implying that SUS304 exhibits high strain-hardening behavior at strains below 0.4. This is also evident in the flow stress curve of Figure 2 obtained via tensile testing. The tensile test of Figure 1 shows the flow stresses at room temperature and almost zero strain; these reference stresses are extrapolated to derive values applicable at higher strain rates and temperatures, with consideration of flow stress information obtained from compression tests performed at various strain rates and temperatures. It should be noted that the flow stresses associated with strains over 0.4 obtained from the compression tests are inaccurate because of losses attributable to barreling, and the fact that higher strain rates are difficult to apply during compression testing. Extrapolations are required to cover the entire range of the state variables during automatic multi-stage cold forging. In the present example, the strain rate attains almost 200/s and the strain reaches 3.0 at the local maxima.

**Figure 5:**
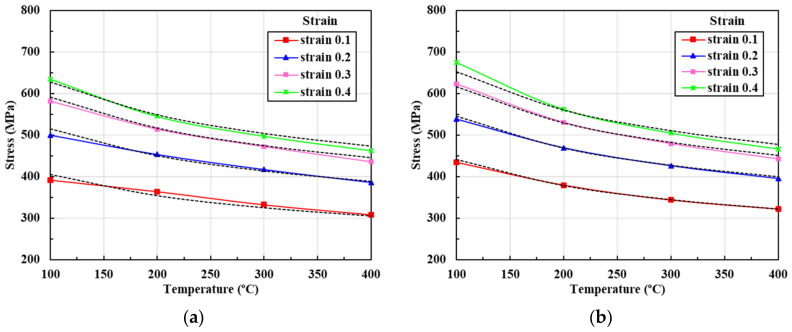

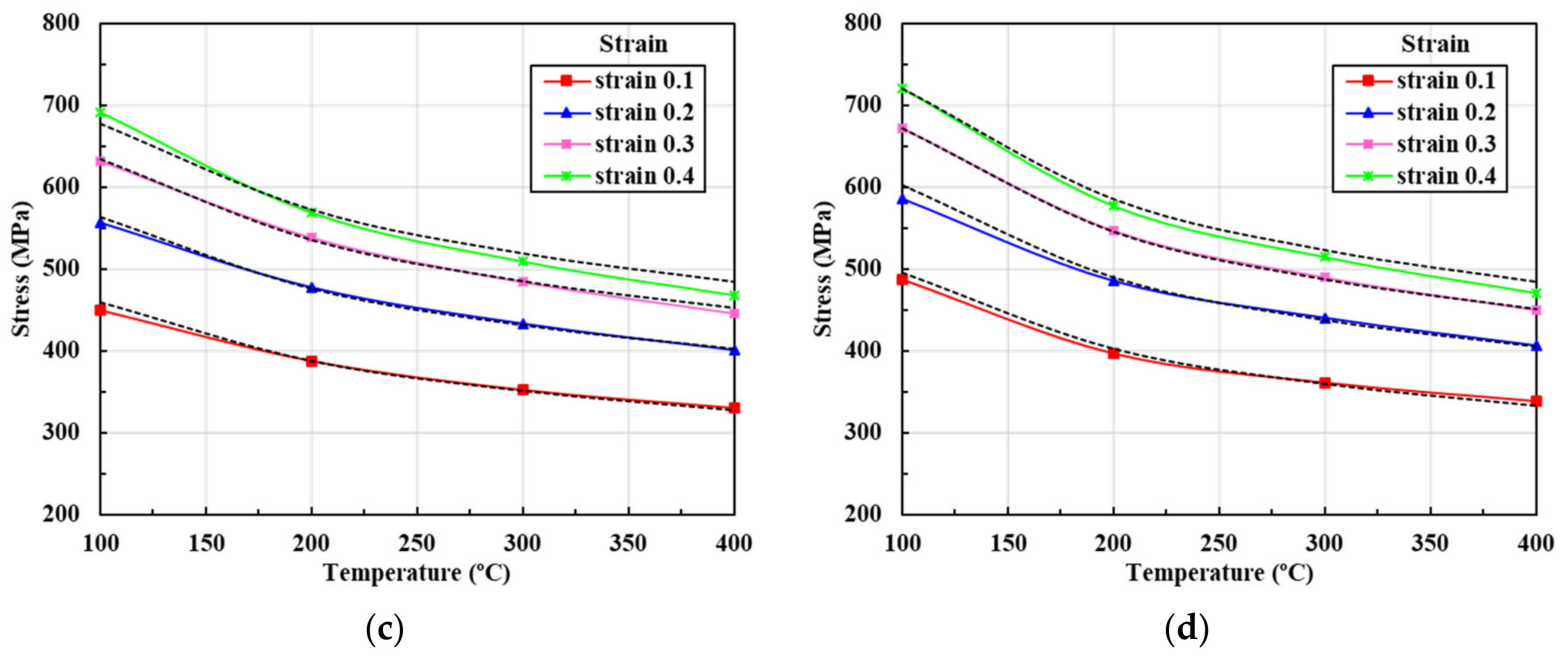
Changes in flow stress with temperature at different sample strains and strain rates: (**a**) 1/s; (**b**) 5/s; (**c**) 10/s; (**d**) 20/s.

The best way to extrapolate flow stress is to derive accurate functions for the appropriate ranges of strain (0.0–0.4), strain rate (1–20/s), and temperature (100–400 °C). Figure 5 shows the changes in flow stress as the temperature rises from 100 to 400 °C on compression testing at a fixed strain rate, indicating that the temperature rise of 300 °C from 100 °C at a strain of 0.3 and strain rate of 20/s decreases the flow stress by about 225 MPa (33%). This explains why plastic deformation is normally evident over consecutive cold forging stages despite the considerable strain-hardening and high contact pressures associated with relatively high-speed, automatic multi-stage cold forging. The flow stress behaviors shown in Figure 5 exhibit similar patterns and we assumed that they could be approximately formulated as follows:

(2) $$\bar{\sigma }/Y_{T}={\left(\frac{1+\beta_{1}T}{1+\beta_{1}T_{0}}\right)}^{\beta_{2}}$$

where $\beta_{1}=0.027$ and $\beta_{2}=-0.242$ are constants derived by minimizing error between the experimental and fitted flow stresses at the tested strains, strain rates, and temperatures. $T_{0}$ is the test room temperature of 20 °C; the right term of Equation (2) becomes unity at room temperature. The maximum temperature-averaged error of the fitted stress with respect to the experimental flow stress was 7.7%. Note that $Y_{T}$ is a function of both the strain and strain rate. The right term is the temperature-weighting function for the sample strains and strain rates shown by the dotted lines.

**Figure 6:**
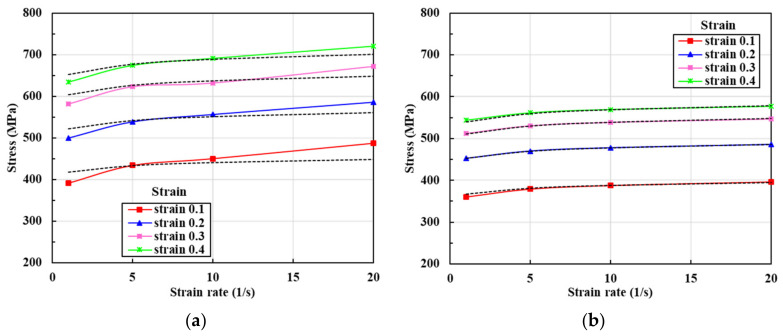

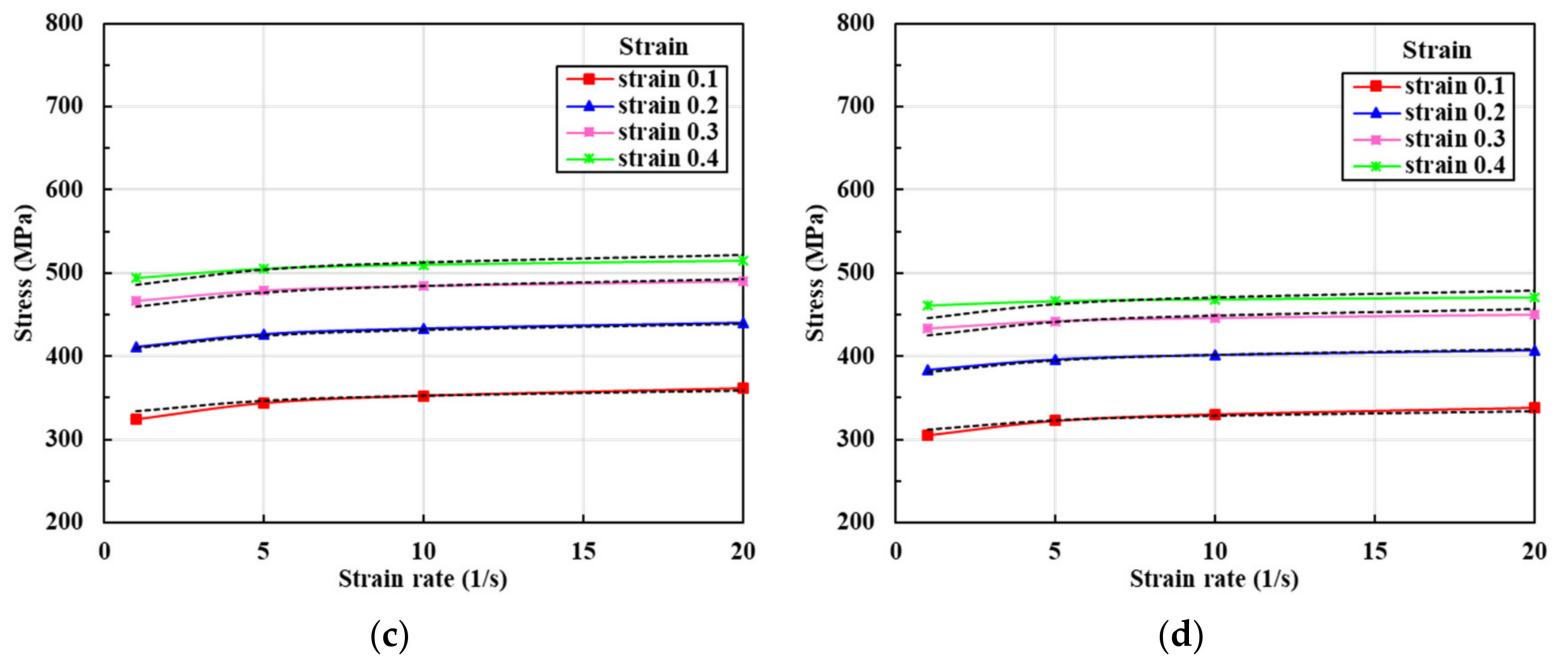
Changes in flow stress by strain rate at various sample strains and temperatures: (**a**) 100 °C; (**b**) 200 °C; (**c**) 300 °C; (**d**) 400 °C.

Figure 6 shows the changes in flow stress by strain rate for temperatures between 100 and 400 °C and strains between 0.0 and 0.4, indicating that the strain rate has a non-negligible effect on flow stress. However, this decreases drastically as the temperature rises. In particular, the strain rate effect of Figure 5 is monotonic, and it would therefore be expected that its effect on flow stress can be expressed by a simple function, as follows:

(3) $$\bar{\sigma }/T_{SR}={\left(1+\gamma_{1}\dot{\bar{\varepsilon }}\right)}^{\gamma_{2}}$$

where $Y_{SR}$, $\gamma_{1}=4.56$ and $\gamma_{2}=0.025$ are calculated to minimize the errors between the experimental and fitted flow stresses at various sample strains, strain rates, and temperatures. Note that $Y_{SR}$ is a function of both strain and temperature. The right term is a strain rate-weighting function for the sample strains and temperatures shown by the dotted lines.

As Equations (2) and (3) are acceptable, the flow stress behaviors of Figure 4 can be derived by multiplying the flow stress at room temperature and a low strain rate (Equation (1)) by the distinct temperature- and strain rate-weighting functions. In other words, the flow stress covering all state variable ranges of interest was assumed to be given by the following closed-form function, in which the variables are separated:

(4) $$\bar{\sigma }=K\left(\bar{\varepsilon }\right){\bar{\varepsilon }}^{n\left(\bar{\varepsilon }\right)}{\left(\frac{1+\beta_{1}T}{1+\beta_{1}T_{0}}\right)}^{\beta_{2}}{\left(1+\gamma_{1}\dot{\bar{\varepsilon }}\right)}^{\gamma_{2}}$$

with all constants already defined.

**Figure 7:**
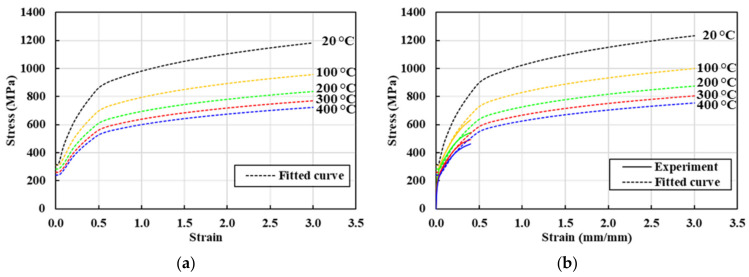

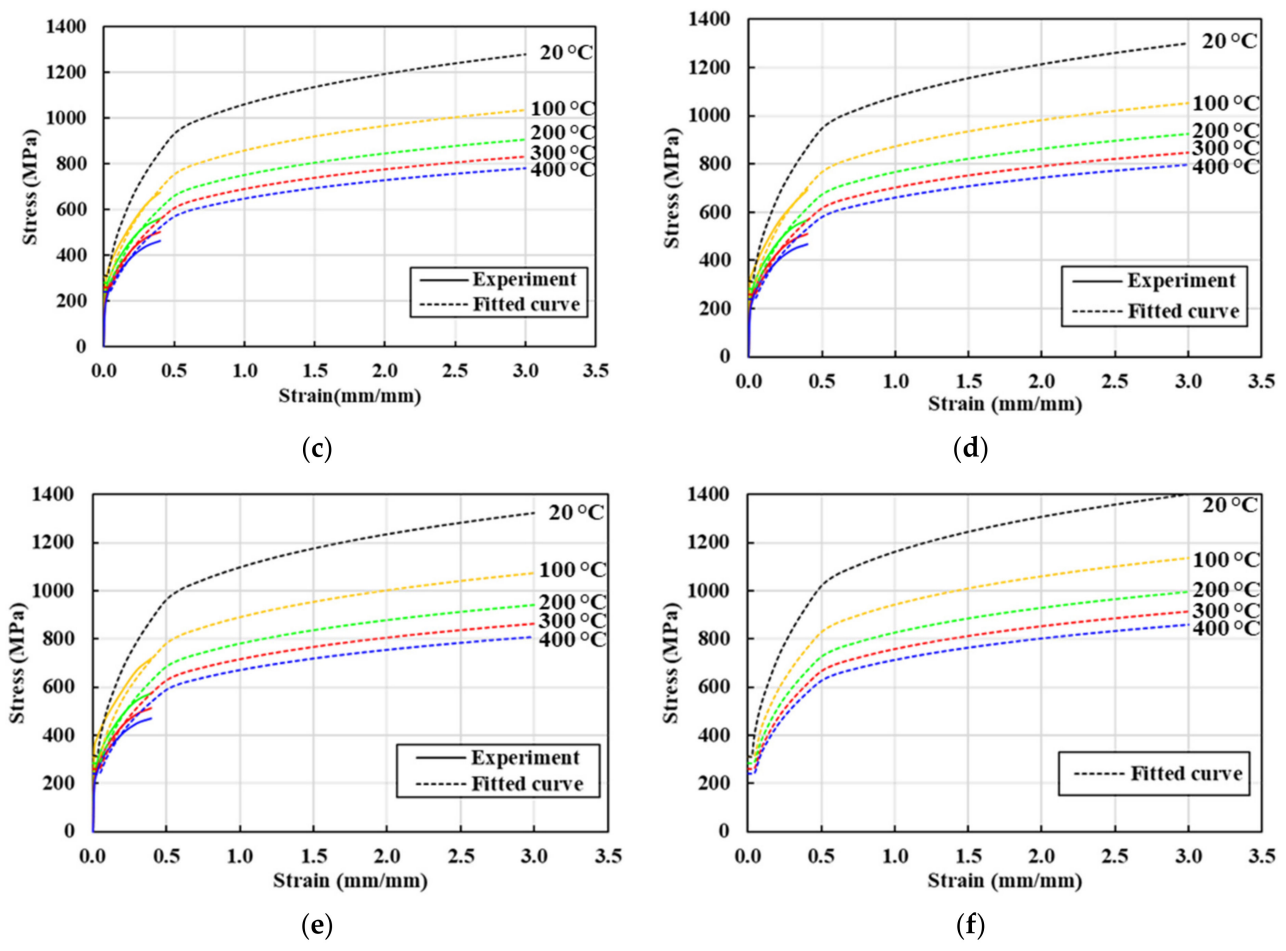
Flow stress curves (dotted lines) covering the entire ranges of the state variables: (**a**) 0/s; (**b**) 1/s; (**c**) 5/s; (**d**) 10/s; (**e**) 20/s; (**f**) 200/s.

The flow stress curves are shown in Figure 7 for the selected sample strain rates and sample temperatures; these cover the entire ranges of the state variables. It is evident that the experimental and fitted flow stresses over the strain range 0.0 to 0.3 are rather close, while those over the strain range 0.3 to 0.4 are quite different (because compression testing is compromised by friction-incurred barreling). The initial yield stresses of Figure 4 were approximated as functions of temperature, as follows:

(5) $$\sigma_{y}\left(T\right)=\sigma_{Y0}+c_{T}\left(T-T_{RT}\right)$$

where $\sigma_{Y0}=314.0$, $c_{T}=-0.18$ and $T_{RT}=20.0$ °C.

Figure 7 shows that the experimental flow stress coverage is small compared to those of the state variables during the real-world process, emphasizing the importance of extrapolation for practical applications. It is also apparent that the strain rate does not greatly affect the flow stress when the temperature is greater than 200 °C, as is also evident in Figure 4. The temperature-averaged errors of the fitted (with respect to the experimental) flow stresses at sample strains of 0.2 and 0.3, sample strain rates of 1, 5, 10, and 20/s, and sample temperatures of 100, 200, 300, and 400 °C were calculated to be 2.0–7.7%, which are simple arithmetic averages of absolute errors of the cases at the same sample strain rates and temperatures, as summarized in Table 2. Note that the sample strains of 0.1 and 0.4 were excluded from the analysis because they are only rough approximations, being compromised by the yield stresses and limitations of compression testing. In other words, the actual flow stress is the maximum of the yield and fitted flow stresses, resulting in some errors at low strains. The compression test tends to underestimate strain-hardening at large strains (above 0.4).

## 3. FEA Predictions and Experimental Data for Automatic, Multi-Stage Cold Forging of an SUS304 Ball-Stud

**Figure 8:**
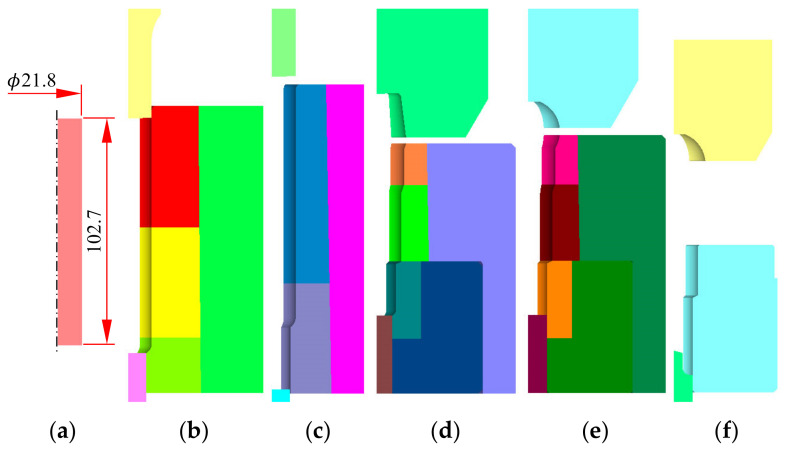
Process design of ball-stud cold forging and die assembly design: (**a**) workpiece; (**b**) stage 1; (**c**) stage 2; (**d**) stage 3; (**e**) stage 4; (**f**) stage 5.

Automatic multi-stage cold forging [23,24,25,26] is used to manufacture fasteners from materials that allow plastic deformation. Beginning a few decades ago, many items manufactured via traditional cold forging are now made via effective, environmentally friendly, automatic multi-stage cold forging, which can handle difficult-to-forge materials including stainless steel and ESWs [7]. Figure 8 shows a forging process design with emphasis on die parts design, exhibiting how the SUS304 ball-stud is automatically cold forged in five stages.

The flow stress of Equation (4) was employed. Note that friction during extrusion varies greatly by position [27], and that the Coulomb friction coefficient was assumed to be a function of strain [28]. To accurately predict how the material temperature affects flow stress, a sophisticated frictional law coefficient is essential. Areas that were severely restricted during extrusion became partially burnt in a previous study [27]. The friction at the die–material interface is very low if the material’s surface is well-lubricated and the surface strain is low. The machine operated at 50 strokes/min. All thermal properties are from [29]. Figure 8 shows a typical tetrahedral finite element mesh [30]. The process was simulated in a fully automatic manner; this greatly facilitates process optimization and die design (including the shrink fit). We used the elastothermoviscoplastic finite element analysis function of a three-dimensional forging module of a commercial metal forming simulator [31,32,33]:

(6) $$\mu =0.02\left(1+\frac{{\bar{\varepsilon }}_{S}}{2}\right)$$

where ${\bar{\varepsilon }}_{S}$ is the effective strain at the contact surface. This deals with any increase in friction caused by the surface strain ${\bar{\varepsilon }}_{S}$ or lubricant degeneration.

**Figure 9:**
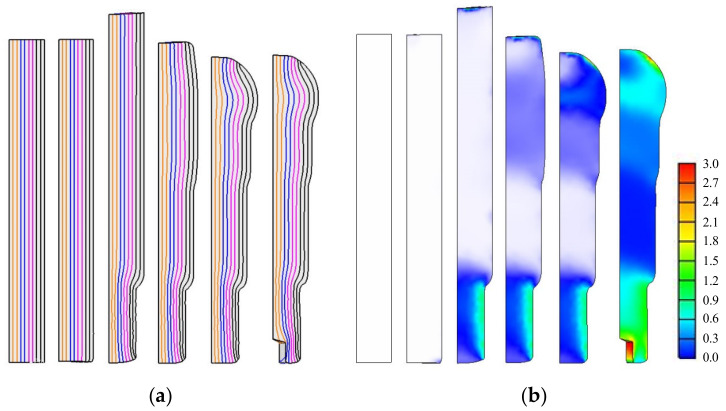
The predicted metal flow lines and effective strains: (**a**) metal flow lines; (**b**) effective strains.

**Figure 10:**
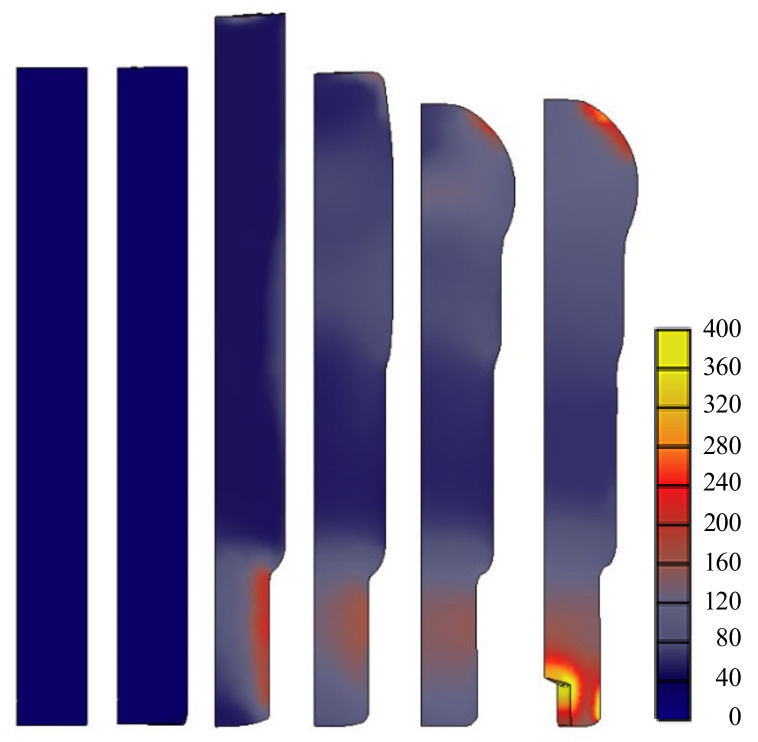
The predicted temperatures.

**Figure 11:**
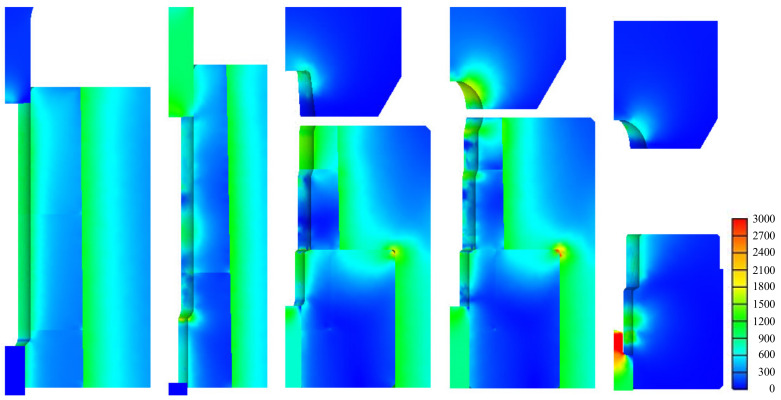
The predicted effective stresses on die parts.

The predictions of the non-isothermal analyses are shown in Figure 9 for the metal flow lines, Figure 10 for temperature, and Figure 11 for the effective stresses on die parts [34]. The predictions showed that the strain and metal flow lines are sound, and that the temperature near the hexagonal hole in the fifth stage is about 440 °C, reducing the effective stress on the die parts at this stage. Note that, at this time, the lower die is weakest and at risk of fatigue fracture as well as severe wear. We used a sliding die [35] supported by a spring at the lower side of the fifth stage to reduce lower die stress.

**Figure 12:**
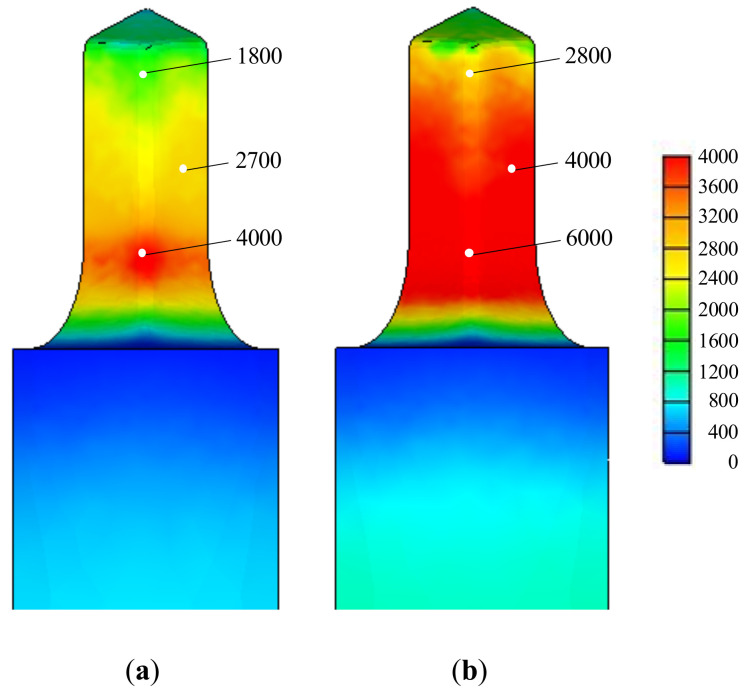
Effective stresses (MPa) on the lower die part at the fifth stage, as calculated via non-isothermal and isothermal analyses: (**a**) Non-isothermal; (**b**) Isothermal.

Isothermal analyses were conducted at room temperature. A comparison of Figure 11 and Figure 12 indicates that the maximum effective stress on the lower die calculated via non-isothermal analysis is 33% less than that derived via isothermal analysis, emphasizing the importance of non-isothermal analysis of the cold forging of difficult-to-forge material. The flow stresses thus derived are acceptable and can be used to evaluate the cold forgeability of high-strength materials, such as stainless steel and ESW material, which exhibit high-level strain-hardening.

**Figure 13:**
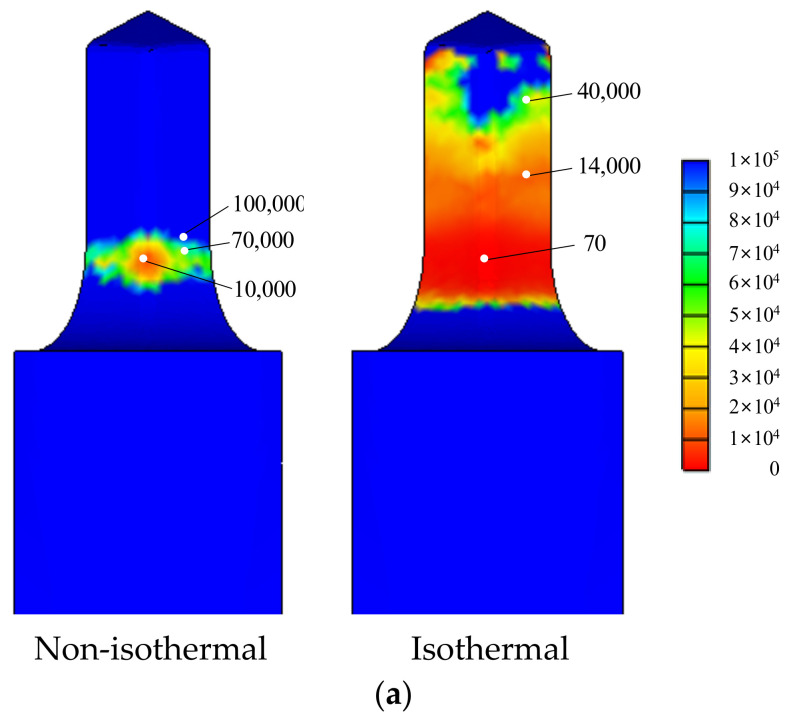

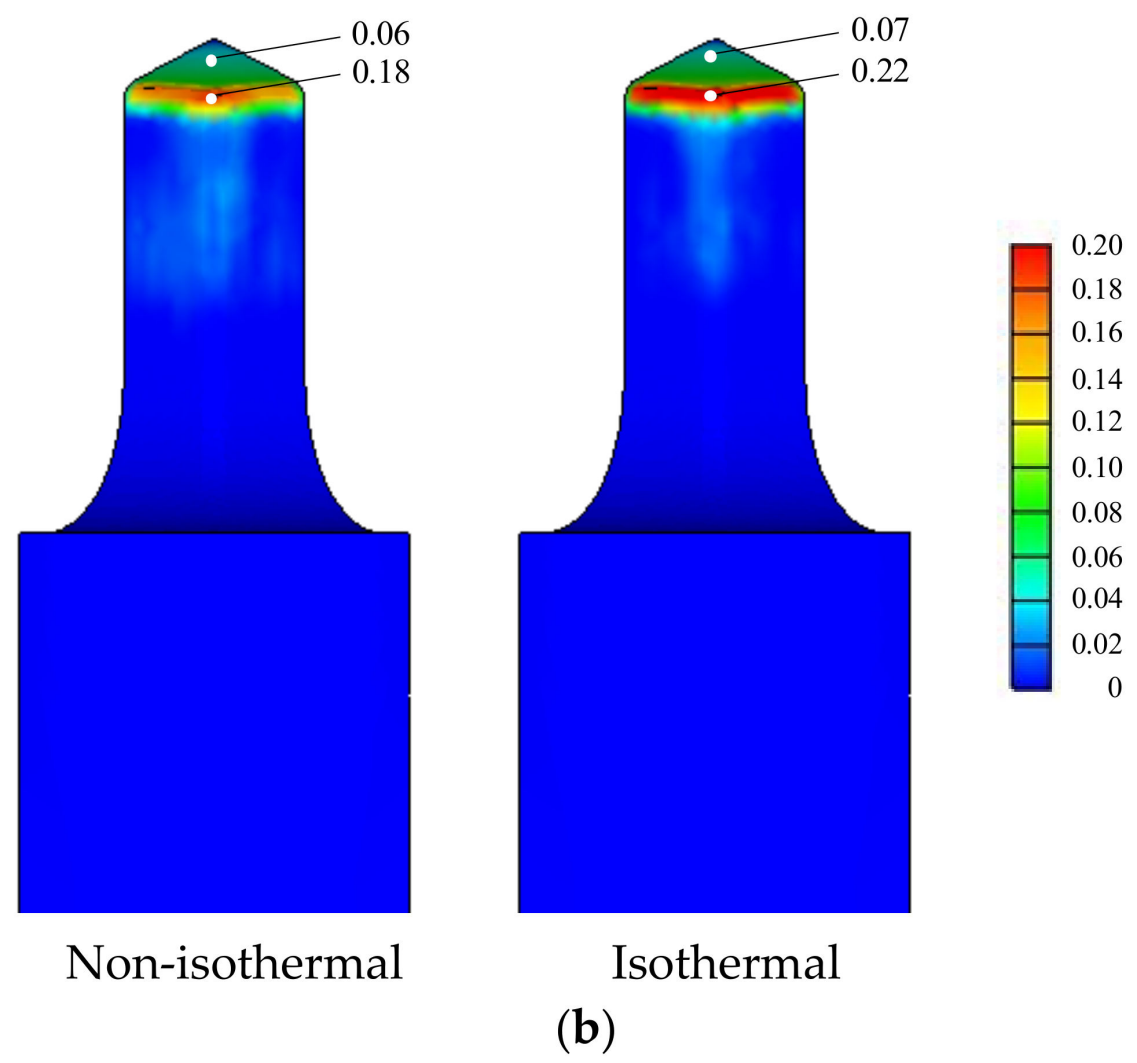
The die fatigue life cycles and wears predicted by non-isothermal and isothermal analyses: (**a**) die fatigue life cycles; (**b**) die wears.

The predicted die fatigue lives are compared in Figure 13a, which were calculated using the die fatigue properties presented by Tanikulu and Karakuzu [18]. The comparison shows that non-isothermal analysis facilitates process design, while isothermal analysis does not. Figure 13b also shows that the predicted die wear index by non-isothermal analysis, which is proportional to frictional energy dissipation at the die surface, is the more promising for the forgeability.

**Figure 14:**
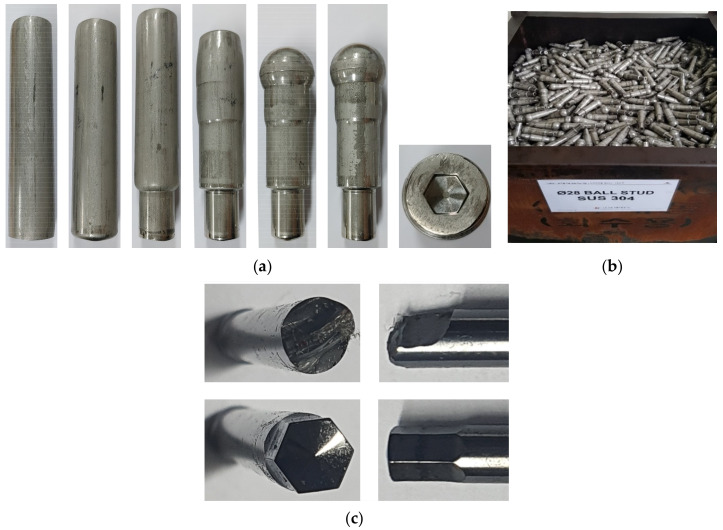
Experiments of SUS304 ball-stud forging: (**a**) experiments; (**b**) a lot of manufacture; (**c**) die failures (upper: fatigue fracture, lower: wear).

Figure 14 shows the experimental data on the SUS304 ball-stud; the geometric properties are good and the hexagonal hole is accurate, as can be seen in Figure 14a. Figure 14b shows a lot of manufacture and Figure 14c shows the die failure cases due to fatigue fracture and die wear, which are compatible with the predictions in Figure 12 and Figure 13.

## 4. Conclusions

We explored the flow stress behaviors of SUS304 using both tensile tests at room temperature and compression tests at temperatures and strains ranging from 100 to 400 °C and 1/s to 20/s, respectively. Cold forging of an SUS304 ball-stud was evaluated with emphasis on the forgeability. The flow behaviors of SUS304 were unique, being characterized by high strength; a high dependence of flow stress on temperature (because of softening at relatively low temperatures); a distinct double strain-hardening capability; and higher strain rate-dependence at low temperatures. This rendered it difficult to model flow stress using traditional methods or fitting schemes. The flow stress of SUS304 at room temperature and low speed was fitted by a double Hollomon model and was characterized by (generally) monotonic changes in response to temperature and strain rate. It was assumed that the separable variable expression of the flow stress could be made at the small strain, which was employed for describing the flow stress covering the whole range of state variables.

Due to the high material strength at the cold forging temperature, and the drastic decrease in flow stress as the temperature increases, the temperature rise inevitably reduces the material strength during forging, as revealed by the flow stresses expressed as a function of strain, strain rate, and temperature over the working ranges of 0.0–3.0, 0.0–200/s and 20–400 °C, respectively. However, there is no practical way to obtain experimental data. We thus extrapolated data using closed-form functions for reliable ranges of the state variables, i.e., a strain of 0.1–0.4, strain rate of 1/s–20/s, and temperature of 20–400 °C. The flow stress was formulated by a multiplication of three separate functions of strain, strain rate and temperature. The experimental and fitted flow stresses showed acceptable agreement.

Notably, the maximum effective stress at the most critical point of the lower die in the fifth stage of the forging process of the SUS304 ball-stud, as predicted by isothermal analysis, was 33% greater than that predicted by non-isothermal analysis, emphasizing the importance of non-isothermal evaluation of flow stresses when exploring the cold forgeability of high-strength materials, including SUS 304 and ESW steel.

We experienced numerically and experimentally an economically successful forging of an SUS304 ball-stud by an automatic multi-stage cold forging machine. Temperature effect emphasized non-isothermal analysis of the cold forging process, which showed positive forgeability in terms of die fatigue fracture as well as die wear in a qualitative way. We showed that fast, consecutive, automatic multi-stage cold forging can be applied to a variety of materials. Temperature control of difficult-to-forge materials lowers costs and pollution and increases efficiency.

## Figures and Tables

**Table 1:** Chemical composition of SUS304.

Component	C	Si	Mn	P	S	Ni	Cr	Cu	Fe
Wt.%	0.037	0.440	0.790	0.034	0.003	8.030	18.010	2.320	Bal.

**Table 2:** Fitted flow stress errors by experimental flow stresses.

$\dot{\varepsilon }$ (1/s)	Temperature (°C)	*ε*	Exp. σ (MPa)	Fitted σ (MPa)	Error (%)	Average Error (%)
1.0	100	0.1	392	376	−4.00	1.98
		0.2	500	493	−1.54	
		0.3	581	577	−0.75	
		0.4	635	645	1.64	
	200	0.1	360	340	−5.59	4.09
		0.2	452	446	−1.44	
		0.3	512	522	1.97	
		0.4	544	584	7.39	
	300	0.1	325	313	−3.74	3.89
		0.2	411	410	−0.44	
		0.3	467	480	2.70	
		0.4	494	537	8.70	
	400	0.1	304	293	−3.57	4.22
		0.2	384	385	0.16	
		0.3	434	450	3.88	
		0.4	461	504	9.28	
5.0	100	0.1	434	403	−7.13	3.08
		0.2	539	528	−2.01	
		0.3	623	618	−0.69	
		0.4	675	692	2.51	
	200	0.1	379	353	−6.97	4.65
		0.2	470	463	−1.55	
		0.3	530	542	2.15	
		0.4	562	606	7.93	
	300	0.1	344	324	−5.71	5.07
		0.2	427	425	−0.43	
		0.3	479	498	3.85	
		0.4	505	557	10.29	
	400	0.1	322	304	−5.53	6.01
		0.2	396	399	0.76	
		0.3	442	467	5.62	
		0.4	466	523	12.15	
10.0	100	0.1	450	410	−8.95	3.64
		0.2	556	537	−3.48	
		0.3	632	629	−0.41	
		0.4	692	704	1.72	
	200	0.1	388	359	−7.45	4.89
		0.2	478	470	−1.48	
		0.3	538	551	2.35	
		0.4	569	616	8.29	
	300	0.1	352	330	−6.43	5.58
		0.2	433	432	−0.31	
		0.3	484	506	4.47	
		0.4	510	566	11.11	
	400	0.1	330	310	−6.25	6.85
		0.2	401	406	1.13	
		0.3	446	475	6.50	
		0.4	468	532	13.53	
20.0	100	0.1	487	417	−14.38	6.61
		0.2	585	546	−6.68	
		0.3	671	640	−4.68	
		0.4	721	716	−0.69	
	200	0.1	397	365	−7.91	5.14
		0.2	485	479	−1.39	
		0.3	547	561	2.57	
		0.4	577	627	8.68	
	300	0.1	361	335	−7.13	6.10
		0.2	440	440	−0.17	
		0.3	490	515	5.12	
		0.4	514	576	11.96	
	400	0.1	339	315	−6.94	7.71
		0.2	407	413	1.53	
		0.3	450	483	7.41	
		0.4	470	541	14.97	
